# Portal Bypass Complicated by Hepatopulmonary Syndrome

**DOI:** 10.1097/PG9.0000000000000155

**Published:** 2021-12-10

**Authors:** Eric J. Monroe, Niviann Blondet, Jeffrey Forris Beecham Chick, Evelyn K. Hsu

**Affiliations:** From the * Department of Radiology, Division of Interventional Radiology, American Family Children’s Hospital & University of Wisconsin, Madison, WI; † Division of Gastroenterology and Hepatology, Seattle Children’s Hospital & University of Washington, Seattle, WA; ‡ Department of Radiology, Division of Interventional Radiology, University of Washington, Seattle, WA.

**FIGURE 1.**
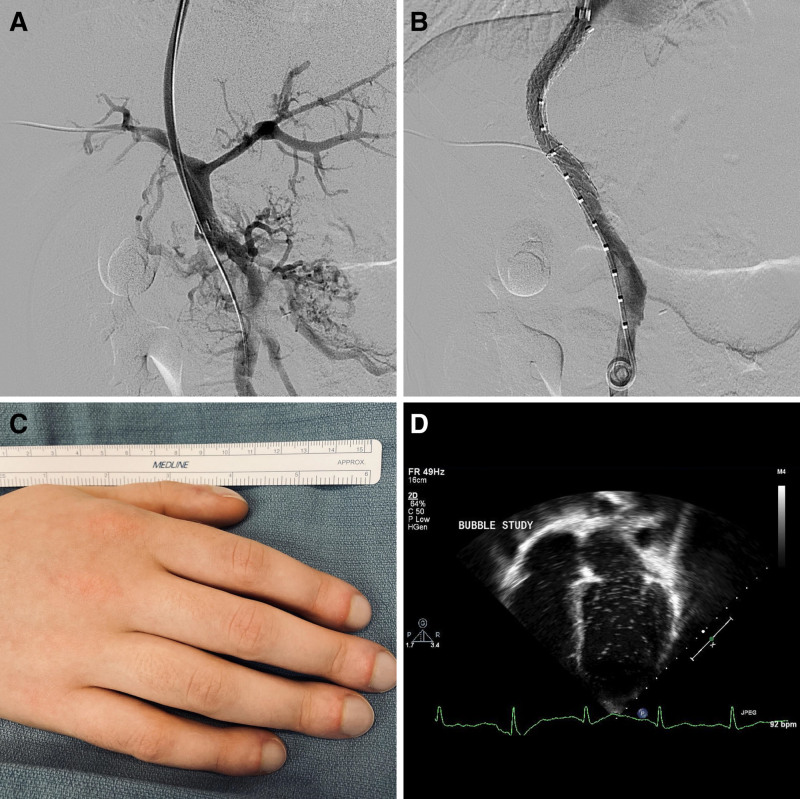
Frontal projection digital subtraction portal venography before (A) and after (B) creation of a transjugular intrahepatic portosystemic shunt. Photograph of the hand (C) demonstrated digital clubbing. Echocardiography with bubble study (D) demonstrated appearance of microbubbles within the left cardiac chambers at 7 cardiac cycles after systemic infusion, confirming intrapulmonary shunting, and the diagnosis of hepatopulmonary syndrome.

At 8 years of age, this patient with cavernous transformation of the portal vein presented with variceal hemorrhage and underwent successful creation of a transjugular intrahepatic portosystemic shunt (TIPS) (Fig. 1A, B). She returned at age 16 years following incidental discovery of TIPS occlusion on yearly surveillance ultrasound. Before induction of anesthesia, she noted to be hypoxemic to 85% and physical examination revealed digital clubbing (Fig. 1C). Following successful TIPS recanalization and angioplasty, she returned for echocardiography with bubble study (Fig. 1D) that confirmed the diagnosis of hepatopulmonary syndrome (HPS). At 1-year follow-up, the TIPS remained patent by Doppler ultrasound and the patient remained asymptomatically hypoxemic in the low 90s at rest with desaturations to the mid 80s with activity.

HPS complicates a small proportion of pediatric liver disease patients. Potential mechanisms include increased production of vasoactive metabolites due to portal hypertension or decreased hepatic clearance due to liver disease or portal bypass. These metabolites lead to pulmonary vasodilation and the formation of intrapulmonary right-to-left shunts (1). The diagnosis is confirmed by echocardiography with bubble study. While TIPS may alleviate hypoxemia in some patients (2), liver transplantation remains the curative treatment. On examination, patients desaturate further in the upright position (orthodeoxia) due to basilar predominance of shunts. In the absence of pulse oximetry or blood gas assessment, digital clubbing may be the first sign of the diagnosis.
